# State-dependent modulation of brain co-activation patterns induced by individually targeted VLPFC stimulation during viewing emotional film clips

**DOI:** 10.3389/fnhum.2026.1824753

**Published:** 2026-05-12

**Authors:** Changyue Hou, Meihua Yan, Haonan Pei, Ting Ye, Sisi Jiang, Hechun Li, Roberto Rodríguez-Labrada, Dezhong Yao, Cheng Luo

**Affiliations:** 1 The Clinical Hospital of Chengdu Brain Science Institute, MOE Key Lab for Neuroinformation, School of Life Science and Technology, University of Electronic Science and Technology of China, Chengdu, China; 2 China-Cuba Belt and Road Joint Laboratory on Neurotechnology and Brain-Apparatus Communication, University of Electronic Science and Technology of China, Chengdu, China; 3 Research Unit of NeuroInformation, Chinese Academy of Medical Sciences, Chengdu, China; 4 Cuban Neuroscience Center, La Habana, Cuba; 5 Department of Radiology, The Fourth People's Hospital of Chengdu, Chengdu, China

**Keywords:** brain dynamics, co-activation pattern, emotional induction, rTMS, state-dependent

## Abstract

Accumulating evidence indicates that repetitive transcranial magnetic stimulation (rTMS) outcomes are state-dependent, with ongoing emotional states influencing neuromodulatory effects. However, how emotional context during stimulation influences subsequent brain functional state organization remains unclear. A total of 99 healthy participants were recruited in this study and allocated to one of three groups: active rTMS with sad film viewing (sad group), active rTMS with neutral film viewing (neutral group), or sham rTMS with sad film viewing (sham group). We combined rTMS during emotional film viewing with co-activation pattern (CAP) analysis to investigate the potential state-dependent effects of rTMS on brain dynamics. The fraction of time, resilience, and transition probabilities of CAPs were calculated to characterize brain dynamics. Four recurring CAPs were identified in this study. Relative to the sham group, participants following stimulation during the sad condition exhibited increased engagement of CAP 2, characterized by deactivations in the frontoparietal network and co-activations of the visual and somatomotor networks, along with decreased engagement of CAP 4, characterized by deactivations in the default mode network and co-activations of the somatomotor and salience networks. In contrast, the neutral condition exhibited attenuated effects. Furthermore, the change in transition probability from CAP 2 to CAP 3 was significantly greater in the sad condition than in the neutral condition. Together, these findings indicate that the effects of rTMS on brain dynamics may be modulated by the emotional context during stimulation. This work offers novel insights into the state-dependent modulation by rTMS and may help inform the optimization of therapeutic outcomes for rTMS interventions.

## 1 Introduction

As a noninvasive neuromodulation approach, repetitive transcranial magnetic stimulation (rTMS) is widely applied in both clinical practice and research to treat a variety of neuropsychiatric diseases, such as Alzheimer’s disease, major depressive disorder, and schizophrenia (Han et al., 2023; Hua et al., 2024; Koch et al., 2022). Although some stimulation protocols can achieve relatively high response and remission rates, accumulating evidence indicates that the efficacy of rTMS across cognitive neuroscience studies and clinical treatments exhibits substantial inter-subject and inter-session variability (Lefaucheur et al., 2020; Ziemann and Siebner, 2015). In addition to stimulation parameters such as dosage, intensity, and protocols, the brain state of an individual during stimulation may also play a critical role in treatment outcomes (Isserles et al., 2011; Li et al., 2016; Pantazatos et al., 2023). In our prior study using the same sample, we focused on static measures of brain activity and demonstrated that TMS during different emotional states can induce state-dependent alterations in brain activity (Hou et al., 2025). However, in current research and clinical practice, brain state is often not considered a key parameter, and its underlying mechanisms remain poorly understood. In particular, previous studies on brain state have largely relied on static measures and have not fully captured the dynamic reconfiguration of large-scale brain networks over time.

The ventrolateral prefrontal cortex (VLPFC) is critically involved in emotion regulation and exhibits functional hypoactivity in patients with various affective disorders (Braunstein et al., 2017; McTeague et al., 2020). Prior research has demonstrated that individuals with depression exhibit a significant reduction in VLPFC gray matter volume (He et al., 2023b). Anatomically, the VLPFC sends axonal projections to the amygdala, and rTMS targeting the VLPFC can further modulate amygdala activity (Kohn et al., 2014). Accordingly, the VLPFC has been identified as a potential target for neuromodulatory interventions in mood, anxiety, and stress-related disorders, with prior studies showing that rTMS targeting the VLPFC enhances the regulation of negative affect in healthy populations, and that anterior VLPFC electrical stimulation yields significant emotional improvement in patients with depression (Cao et al., 2021; Rao et al., 2018; Sydnor et al., 2022). In summary, these findings indicate that targeted stimulation of the VLPFC may help alleviate clinical symptoms in individuals with emotional disorders. Sadness, as a basic emotion, typically arises in response to negative life events or experiences of loss (Zaid et al., 2021). Effective regulation of sadness and other negative emotions is essential for maintaining psychological well-being, whereas prolonged or dysregulated negative emotional experiences are considered important risk factors for psychiatric disorders (Kovacs et al., 2016; Woody and Gibb, 2015). Film clips are considered one of the most powerful and effective paradigms for eliciting emotional responses (Deng et al., 2017; Ge et al., 2019). Owing to their dynamic and multimodal nature, they more closely approximate emotional experiences encountered in real-world contexts (Uhrig et al., 2016). In line with this, several standardized and validated databases of affective video stimuli have been developed, including the Chieti Affective Action Videos database (Di Crosta et al., 2020; La Malva et al., 2021), the Emotional Movie Database (Carvalho et al., 2012), and the Visual Affective Stimulus Database (Li et al., 2022), which provide well-characterized stimuli with normative ratings. Accordingly, applying rTMS during sad film viewing allows for the investigation of state-dependent neuromodulatory effects on emotion regulation within an ecologically valid emotional context.

As a data-driven approach, co-activation pattern (CAP) analysis offers a robust and sensitive method of characterizing dynamic functional connectivity in the brain (Liu et al., 2013). CAP analysis has been widely applied to investigate transient brain network dynamics in both neuropsychiatric disorders and basic neuroscience research. Previous studies have shown that higher depressive symptom severity is linked to greater frequency and longer dwell time of CAPs dominated by the DMN (Goodman et al., 2021), and transient dynamic abnormalities in the brain’s functional activity have also been reported in individuals with autism spectrum disorder (Li et al., 2024). Moreover, in our previous work combining simultaneous transcranial direct current stimulation and functional magnetic resonance imaging, we demonstrated that the central executive network–targeted transcranial direct current stimulation significantly modulates brain dynamics (Li et al., 2025).

The present study applied rTMS during emotional film viewing and employed CAP analysis to quantify changes in the distribution of functional brain states before and after stimulation. This approach aimed to characterize the state-dependent modulatory impacts of rTMS on brain dynamic organization within a sadness-related emotional context. A clearer understanding of the state-dependent effects of rTMS could facilitate more informed and systematic applications of neuromodulation across different cognitive and emotional states and offer insights relevant to the refinement of neuromodulation-based therapeutic strategies.

## 2 Methods

### 2.1 Participants

One hundred and six healthy, right-handed young adults were recruited from the University of Electronic Science and Technology of China. Participants were randomly allocated to one of three groups. All subjects reported normal or corrected-to-normal vision and no substance use, contraindications to magnetic resonance imaging and rTMS, or history of neurological or psychiatric illness. Seven participants withdrew due to rTMS-related discomfort or scheduling issues. Before and after the intervention, participants completed a battery of measures, including the Self-Rating Depression Scale (SDS), the Self-Rating Anxiety Scale (SAS), and the Interpersonal Reactivity Index–C (IRI-C). The IRI-C contains four subdomains: perspective taking (PT), fantasy (FS), empathic concern (EC), and personal distress (PD).

### 2.2 Experimental design

**Figure 1:**
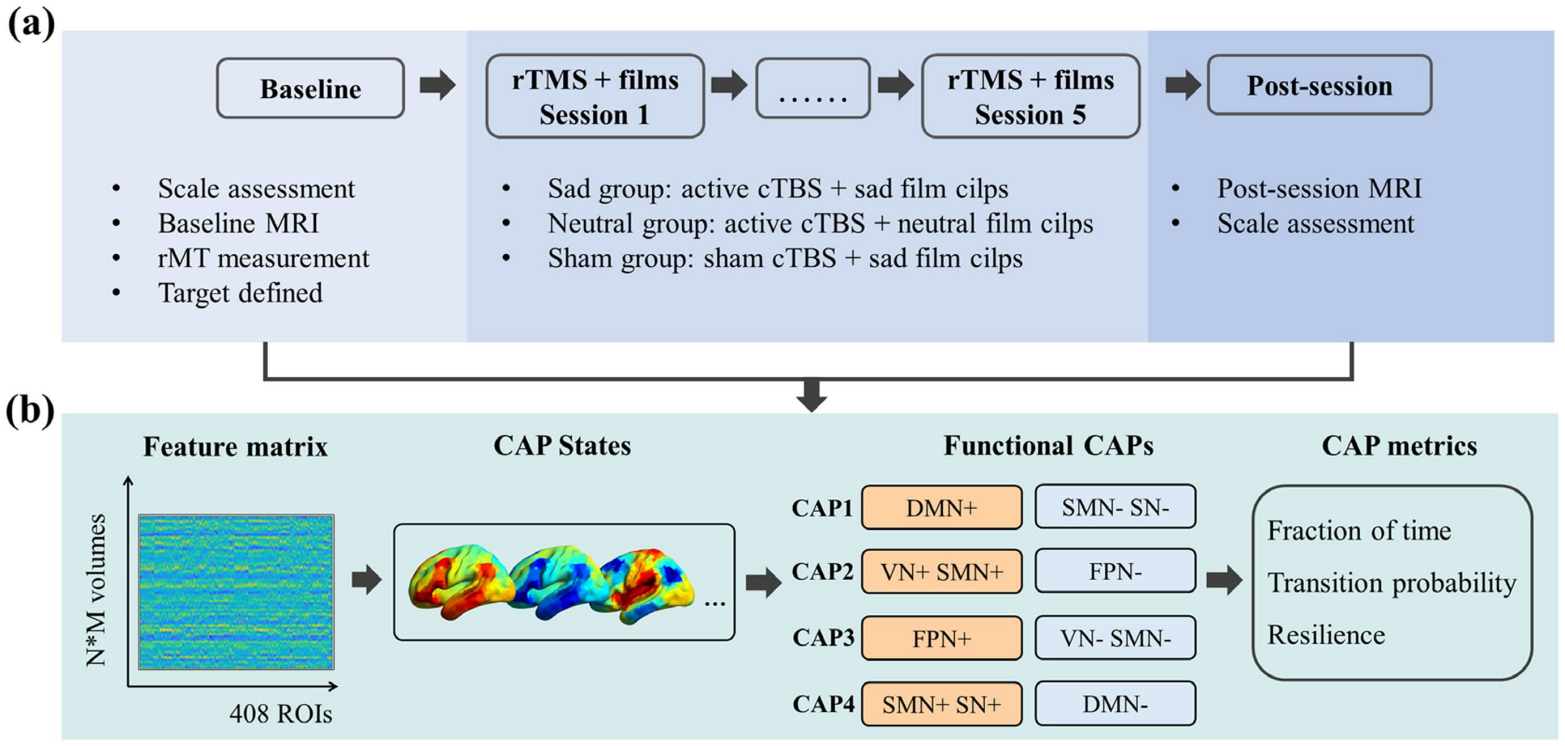
Experimental procedure and data analyses. **(a)** Experimental procedure. The experiment spanned six consecutive days. **(b)** Schematic overview of the co-activation pattern (CAP) analysis. N indicates the number of participants, and M indicates the number of volumes for each participant.

As depicted in Figure 1A, the study protocol spanned six consecutive days. On Day 1, participants completed baseline behavioral assessments and underwent a baseline MRI scan. Over the following 5 days, each participant received a daily 40-s continuous theta-burst stimulation (cTBS) protocol. Immediately after the final stimulation session on Day 6, participants underwent a post-session MRI scan and completed the same behavioral scales as those obtained at baseline. Throughout the five stimulation days, participants viewed film clips during each cTBS session. Stimulus film clips were sourced from the New Standardized Emotional Film Database for Asian Culture (Deng et al., 2017). These clips included sad film clips aimed at evoking negative affect and neutral film clips that served as control stimuli, eliciting neither sadness nor other strong emotional responses. Each emotion was presented through 5 clips, each lasting more than 150 s. Based on the film type and stimulation condition, participants were randomly allocated into three groups: a neutral group receiving active cTBS during viewing neutral film clips, a sad group receiving active cTBS during viewing sad film clips, or a sham group receiving sham cTBS during viewing sad film clips. Neuro-navigated cTBS was delivered during the last 40 s of every film clip via E-prime 3.0 (Psychology Software Tools Inc., Sharpsburg, USA). Following TMS stimulation and film viewing, participants were asked to rate their emotional experiences elicited by the films. Specifically, ratings were provided for sadness, pleasure, arousal, and dominance on a 1–9 scale, with 1 indicating the lowest level and 9 indicating the highest level of each dimension.

### 2.3 rTMS protocol

Active stimulation sessions were delivered using a Magstim® Super Rapid2 stimulator with a 70-mm figure-8 coil. Sham stimulation was administered with a Magstim sham coil. The coil generates a mechanical clicking sound with each pulse, closely resembling that of the active stimulation and making the two conditions perceptually indistinguishable. This study employed a single-blind design, in which participants were unaware of their stimulation condition. Neuronavigation was performed using Brainsight, into which individual T1-weighted images and stimulation target coordinates were imported. On the first day, resting motor threshold (rMT) was determined by recording surface electromyography from the right abductor pollicis brevis while delivering single-pulse stimulation over the left primary motor cortex (M1). rMT was defined as the minimum stimulation intensity that evoked motor-evoked potentials exceeding 50 μV in at least 5 out of 10 consecutive trials. Stimulation was applied at 80% of each participant’s rMT. The study used a cTBS protocol consisting of 50-Hz triplets repeated every 200 ms for a duration of 40 s, delivering a total of 600 pulses per day.

### 2.4 Data acquisition and TMS site of stimulation localization

MRI data were collected on a 3.0-T scanner (Discovery MR750, GE, USA) equipped with a 32-channel head coil. High-resolution anatomical images were obtained using a T1-weighted magnetization-prepared rapid gradient echo (MPRAGE) sequence [repetition time (TR) = 5.964 ms, echo time (TE) = 1.972 ms, field of view (FOV) = 256 × 256 mm^2^, flip angle = 9^°^, slice thickness = 1 mm, matrix size = 256 × 256, and 156 volumes]. T1 images were imported into BrainSight Software for neuronavigation. Functional images, including resting-state and emotional face-matching task fMRI, were acquired using a standard echo-planar imaging (EPI) sequence (TR = 2000 ms, TE = 30 ms, FOV = 240 × 240 mm^2^, flip angle = 90^°^, slice thickness = 3.4 mm, matrix size = 64 × 64, slices = 39). For the resting-state scan, 255 volumes were collected over 510 s. During this scan, participants were instructed to keep their eyes closed, remain awake, and let their thoughts wander.

At baseline, an emotional face-matching fMRI task with a standard block-design paradigm was conducted to identify the individualized right VLPFC stimulation site. In each trial, participants viewed a reference image (facial expression or oval shape) above two options and indicated which option matched the reference. The task comprised 12 blocks (five emotional-face and two oval-shape trials per block), lasted 256 s, and produced 128 volumes.

For the face-matching task images, we performed slice timing, realignment, spatial smoothing, and co-registration of structural images and functional images. After preprocessing, task-state data were analyzed using a general linear model in the statistical parametric mapping (SPM12),^1^ with emotional-face and oval-shape conditions convolved with the canonical hemodynamic response function (HRF) and six motion parameters entered as nuisance regressors. Individual-level beta maps were examined to locate the voxel exhibiting the strongest activation within the right VLPFC, which was subsequently selected as the stimulation target (individual TMS target coordinates are shown in Supplementary Figure S1).

### 2.5 Resting-state fMRI preprocessing and co-activation pattern analysis

fMRI data were preprocessed in MATLAB using the Data Processing Assistant for Resting-State fMRI (DPARSF) toolbox.^2^ The initial five volumes of each run were removed. Resting-state fMRI data then underwent slice-timing correction, realignment for head motion, spatial normalization to the Montreal Neurological Institute space with a 3 × 3 × 3 mm^3^ voxel resolution, and spatial smoothing with an 8-mm full-width at half maximum Gaussian kernel. The participants with head motion greater than 2 mm or/and 2° were removed. Nuisance regression included the Friston-24 motion parameters, white matter and cerebrospinal fluid signals, global signal regression, as well as linear trends. The time series were band-pass filtered at 0.01–0.08 Hz.

Co-activation pattern analysis was used to detect recurring brain states across time frames that exhibit similar spatial co-activation. The BOLD signals within the 408 ROIs (400 cortical parcels based on Yeo’s 7-network parcellation and 8 subcortical parcels from the AAL atlas) (Schaefer et al., 2018; Tzourio-Mazoyer et al., 2002) were extracted from the preprocessed fMRI data and subsequently normalized using a *z*-score across time frames to represent the relative activation magnitude changes. For each subject, this procedure generated a two-dimensional normalized BOLD matrix (number of time frames × number of ROIs) in each scan. In this study, all subjects’ two scans (pre-session and post- session) were concatenated to derive the CAP patterns, yielding a final two-dimensional BOLD matrix of 49,500 × 408 (49,500 = 250 × 99 × 2). K-means clustering was then applied to identify recurring co-activation patterns. The number of clusters (*K*) was set to range from 2 to 11 (step size = 1), with 100 repetitions performed for each *K*. Correlation distance (1 minus the Pearson correlation coefficient) was used to assess the spatial similarity between time frames. Averaged maps for each CAP state were further normalized relative to the within-cluster standard deviation to yield standardized CAP maps. The data analyses are illustrated in Figure 1b.

Based on the assignment of time frames from the clustering analysis, several CAP-related temporal dynamics metrics were computed: (i) Fraction of time, the proportion of time points assigned to a given CAP state *i*, representing the percentage of time the brain spends in that specific network state; (ii) Transition probability, the likelihood of switching from state *i* to state *j*. If a total of N transitions originate from state *i*, and m of them transition to state *j*, then the transition probability from *i* to *j* is m/N; (iii) Resilience, the self-transition probability of state *i*. Pearson correlation was used to further assess spatial similarity across CAPs.

### 2.6 CAP definition

We then parcellated 408 ROIs into seven networks, including the dorsal attention network (DAN), ventral attention network (VAN; also referred to as the salience network, SN), limbic network, visual network (VN), somatomotor network (SMN), frontoparietal network (FPN), and default mode network (DMN). Following temporal clustering, the CAPs were further defined (Liu et al., 2022). Each time frame for every subject was assigned to a specific CAP state and labeled accordingly. Time frames sharing the same label were then averaged within each subject to generate individual CAP maps (N × 408 ROIs, where N = number of CAPs). A Wilcoxon signed-rank test was conducted across all participants, and group CAPs were identified. Next, spatial clustering based on Euclidean distance was applied to estimate the z-score distribution of each CAP. To obtain three distinct activation states (high, medium, and low), the k-means clustering was performed 500 times with *K* = 3. Based on the ROI mask, the ROI-level activations were assigned to all voxels within each ROI. According to the network affiliation of each voxel in the network parcellation, the corresponding network-level significant co-activations and deactivations were obtained. Functional CAPs were then defined based on the peak ratio of network-level activations.

### 2.7 Statistical analysis

A one-way analysis of covariance (ANCOVA) was conducted to assess group differences in temporal dynamics changes (post-stimulation minus pre-stimulation), with age, sex, and education level included as covariates. *Post hoc* pairwise comparisons were conducted using Bonferroni correction, while no correction for multiple comparisons was applied across different temporal dynamic metrics.

Finally, the associations between CAP temporal metrics and behavioral scores were evaluated using Pearson partial correlation analyses, controlling for age, sex, and educational level. No correction for multiple comparisons was performed across correlation analyses, and the results should be interpreted with appropriate caution.

## 3 Results

### 3.1 Demographic and behavioral characteristics

In the end, 99 participants were retained for this analysis. Pre-session scale data were available for all participants, whereas post-session scale data were available for 32, 28, and 20 participants in the neutral, sad, and sham groups, respectively. No significant differences in age, sex, educational level, and baseline scale scores were observed across the three groups. Table 1 provides a summary of the detailed participant characteristics.

**Table 1:** Demographic variables of the three groups^a^.

Variables	Group^b^			Statistics
	Sad (*n* = 39)	Neutral (*n* = 40)	Sham (*n* = 20)	
Age (years)	23.3 ± 1.3	23.1 ± 1.1	22.9 ± 1.6	0.404^c^
Education (years)	17.3 ± 1.2	17.3 ± 1.0	16.9 ± 1.5	0.303^c^
Male/Female	18/21	20/20	8/12	0.764^d^
Handedness (self-reported)	Right	Right	Right	–
IRI-C	73.3 ± 7.6	72.4 ± 9.2	71.8 ± 6.2	0.765^c^
SDS	31.0 ± 6.6	31.0 ± 7.0	31.5 ± 8.3	0.954^c^
SAS	30.1 ± 6.7	30.0 ± 6.8	30.5 ± 7.4	0.958^c^
Post IRI-C	70.6 ± 6.1	71.9 ± 9.9	71.0 ± 4.5	0.890^c^
Post SDS	31.5 ± 5.8	28.9 ± 7.0	30.2 ± 9.3	0.277^c^
Post SAS	29.8 ± 5.4	28.1 ± 7.1	29.2 ± 6.6	0.489^c^

^a^Data are expressed as mean ± S. D. ^b^“Sad” refers to participants who received active cTBS during viewing sad film clips; “Neutral” refers to those who received active cTBS during viewing neutral control clips; and “Sham” refers to participants who received sham cTBS while watching the same sad clips. ^c^The *p*-value was obtained using a one-way analysis of variance. ^d^The *p*-value was obtained using a two-tailed Pearson *χ*^2^ test.

In addition, a one-way analysis of variance revealed a significant group effect on the mean sadness and arousal ratings across the 5 days (*p* < 0.001). Post hoc pairwise comparisons with Bonferroni correction showed that the sad group and sham group exhibited higher ratings than the neutral group, with no difference between the sad group and sham group (see Supplementary material and Supplementary Figure S2 for details). These findings support the validity of the emotional induction.

### 3.2 Co-activation patterns and brain states

**Figure 2:**
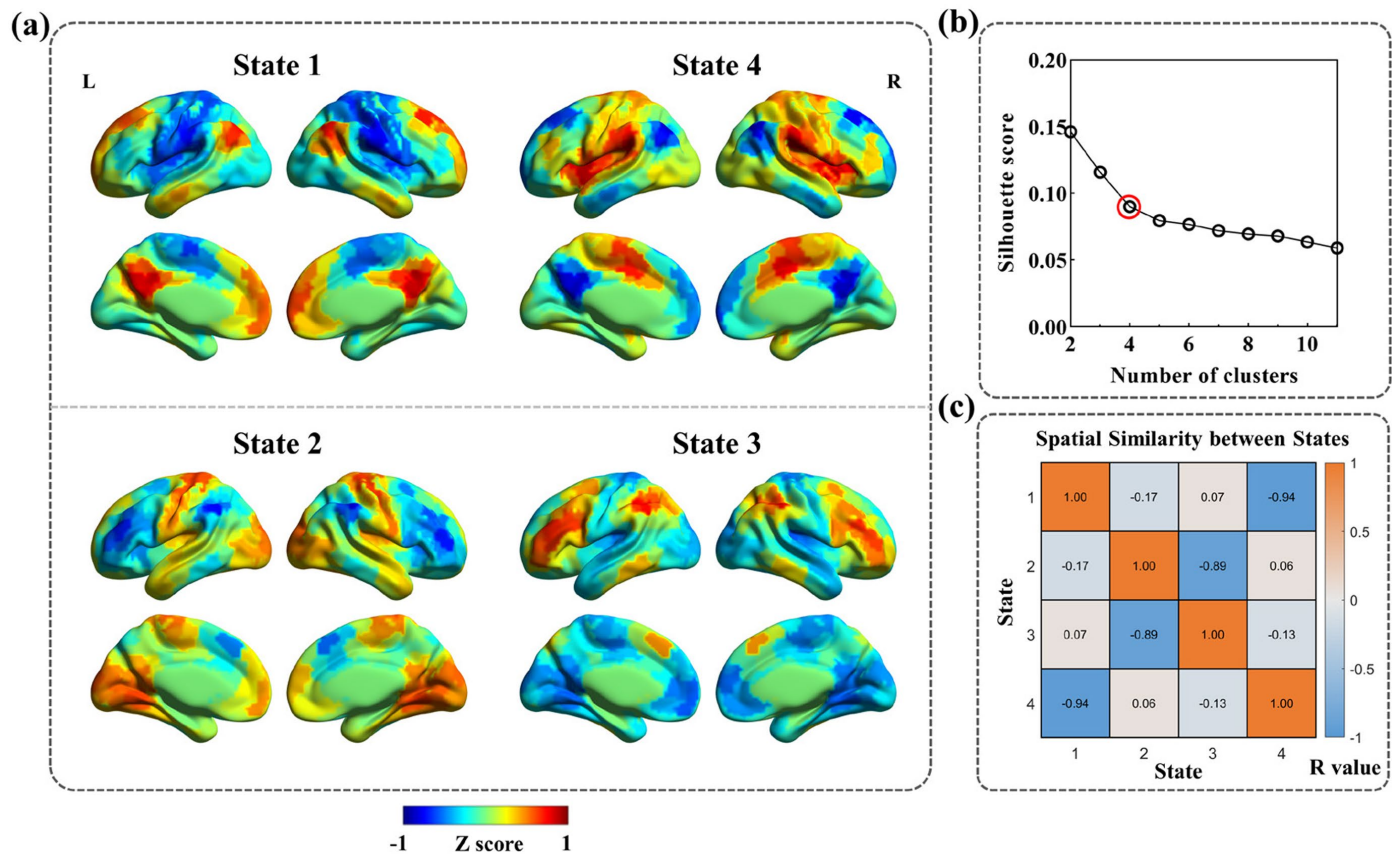
CAP state. **(a)** Surface rendering of CAP states for visualization and characterization of state-space properties. Four states were identified and normalized at the group level, with values expressed as *z*-statistics. Red indicates a relatively stronger activation, whereas blue indicates a relatively stronger. **(b)** The clustering curve. Silhouette score was calculated from *K* = 2 to *K* = 11 with step length = 1, and *K* = 4 was chosen. **(c)** Group average state spatial similarity matrix across four states, quantified using the Pearson correlation.

Co-activation patterns were derived across all participants using temporal k-means clustering, and four CAP states were ultimately identified based on silhouette scores (Figures 2a,b). Figure 2c illustrates the spatial similarity among CAP states, quantified using pairwise Pearson correlation coefficients. As shown, State 1 and State 4 displayed the strongest negative spatial correlation, whereas State 2 and State 3 also exhibited markedly negative correlations in their spatial patterns.

**Figure 3:**
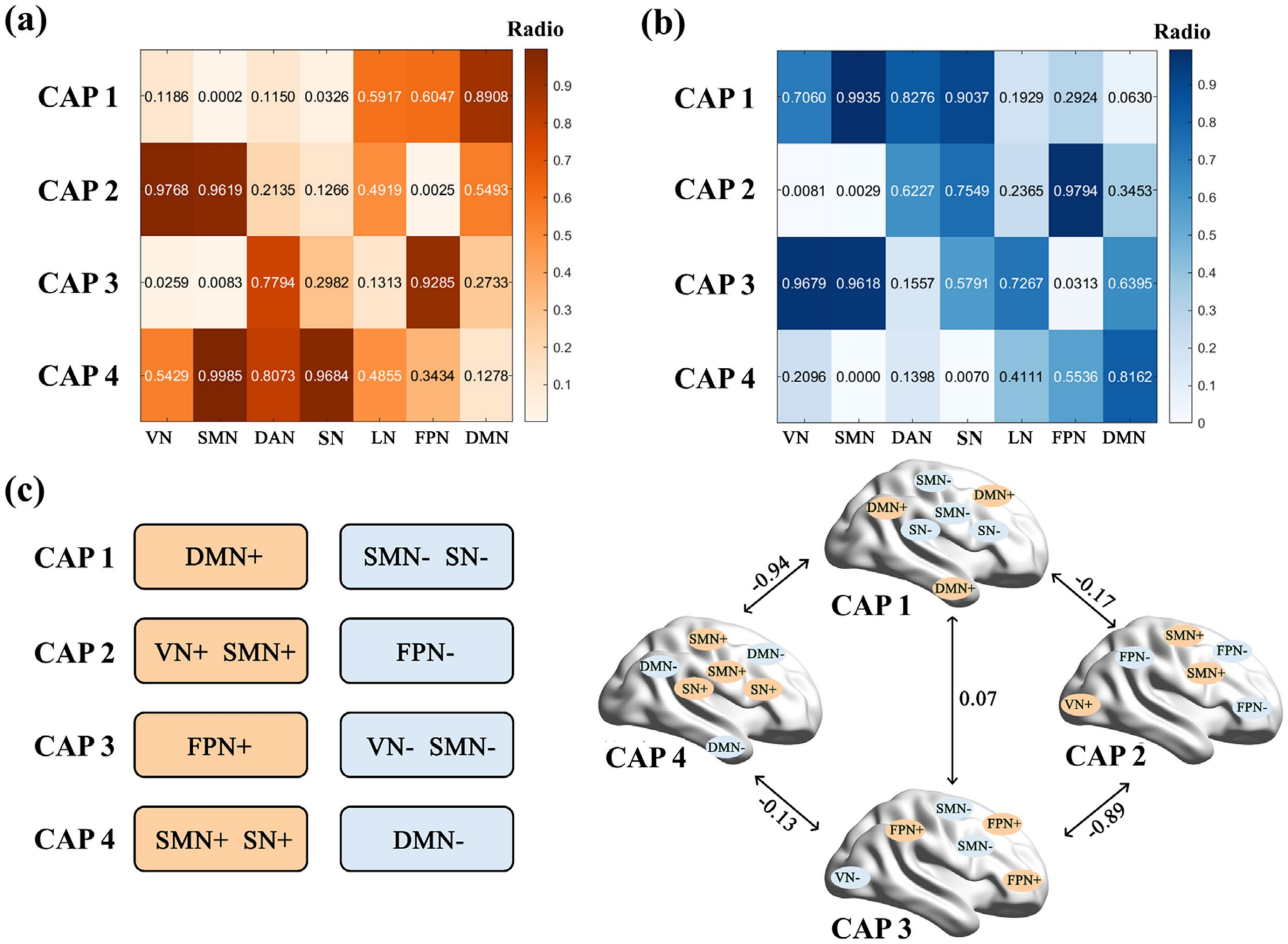
Functional CAPs. **(a)** Ratio of peak activations within each network across different CAPs. The matrix displays the average proportion of co-activations for each network. Each element in the matrix indicates the ratio of peak co-activation voxels to all voxels in the network. **(b)** The matrix displays the average proportion of deactivations for each network. Each element in the matrix indicates the ratio of peak deactivation voxels to all voxels in the network. **(c)** Network activation patterns characterizing the four CAPs. The “+” and “−” symbols indicate co-activation and deactivation, respectively.

Based on the peak co-activations (indicated by “+”) (Figure 3a) and deactivations (indicated by “−”) (Figure 3b) observed within each network, the four CAP states were further characterized as four functional CAPs (CAP 1 DMN + SMN − SN−, CAP 2 VN + SMN + FPN–, CAP 3 FPN + VN − SMN−, and CAP 4 SMN + SN+DMN−) (Figure 3c). Specifically, CAP 1 demonstrated a high proportion of co-activations within the DMN (proportion = 0.89), accompanied by deactivations in the SMN (proportion = 0.99) and SN (proportion = 0.90). CAP 2 was characterized by predominant co-activations within the VN (proportion = 0.98) and SMN (proportion = 0.96), and deactivations in the FPN (proportion = 0.98). CAP 3 indicated a high proportion of co-activations in the FPN (proportion = 0.93), along with deactivations in the VN (proportion = 0.97) and SMN (proportion = 0.96). CAP 4 primarily involved co-activations in the SMN (proportion = 0.999) and SN (proportion = 0.97), together with DMN deactivations (proportion = 0.82).

### 3.3 Differences in temporal dynamic metrics among the three groups

**Figure 4:**
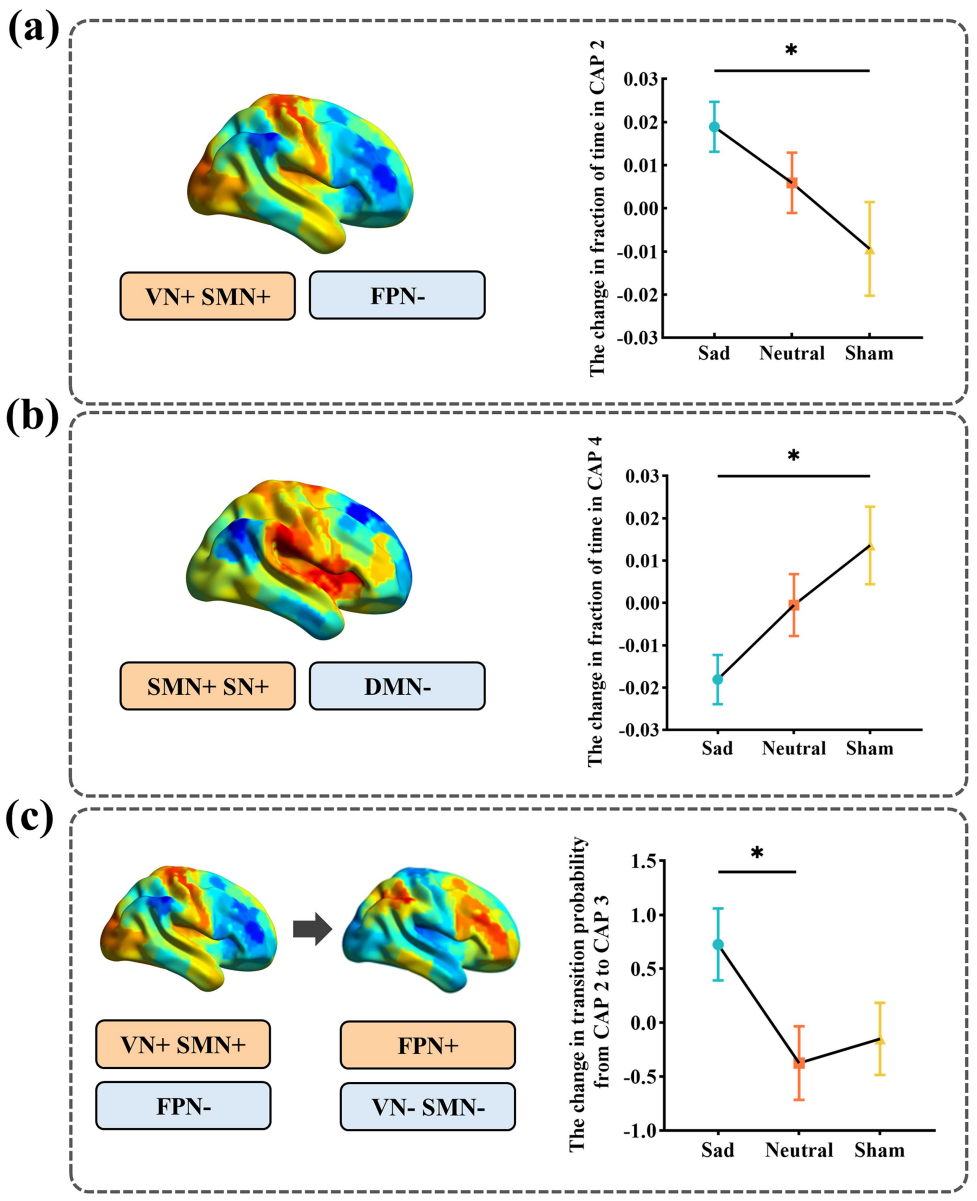
Group differences in temporal metrics among the three groups. **(a)** Group differences in the change in fraction of time in CAP 2 among the three groups. **(b)** Group differences in the change in fraction of time in CAP 4 among the three groups. **(c)** Group differences in the change in transition probability from CAP 2 to CAP 3 among the three groups. Points represent group means, and error bars indicate the standard error of the mean (SEM). Multiple between-group and pairwise comparisons were conducted by Bonferroni’s *post hoc* test. *p < 0.05.

A significant group effect was observed for changes in CAP 2 fraction of time by ANCOVA (*F*_(2, 93)_ = 3.617, *p* = 0.031). *Post hoc* pairwise comparisons with Bonferroni correction revealed that the sad group exhibited a significantly greater increase in CAP 2 fraction of time compared with the sham group (mean difference = 0.031, *p* = 0.026, 95% CI [0.003, 0.060]). Neither the sad versus neutral comparison nor the neutral versus sham comparison reached statistical significance (*p* = 0.565 and *p* = 0.336, respectively) (Figure 4a). Similarly, a significant group effect was also observed for changes in CAP 4 fraction of time (*F*_(2, 93)_ = 5.332, *p* = 0.006). Post hoc pairwise comparisons with Bonferroni correction demonstrated that the sad group showed a significantly greater decrease in CAP 4 fraction of time compared with the sham group (mean difference = −0.035, *p* = 0.007, 95% CI [−0.062, −0.008]). No significant differences were observed between the sad and neutral groups or between the neutral and sham groups (*p* = 0.122 and *p* = 0.440, respectively) (Figure 4b).

A significant group effect was observed for changes in transition probability from CAP 2 to CAP 3 (*F*_(2, 93)_ = 4.727, *p* = 0.011). Post hoc pairwise comparisons with Bonferroni correction revealed a significantly greater increase in transition probability from CAP 2 to CAP 3 in the sad group than in the neutral group (mean difference = 1.327, *p* = 0.009, 95% CI [0.266, 2.388]), whereas no significant differences were observed between the other group pairs (*p* = 0.329 and *p* = 1.000, respectively) (Figure 4c).

### 3.4 Relationships between temporal dynamics metrics and behavioral measures

**Figure 5:**
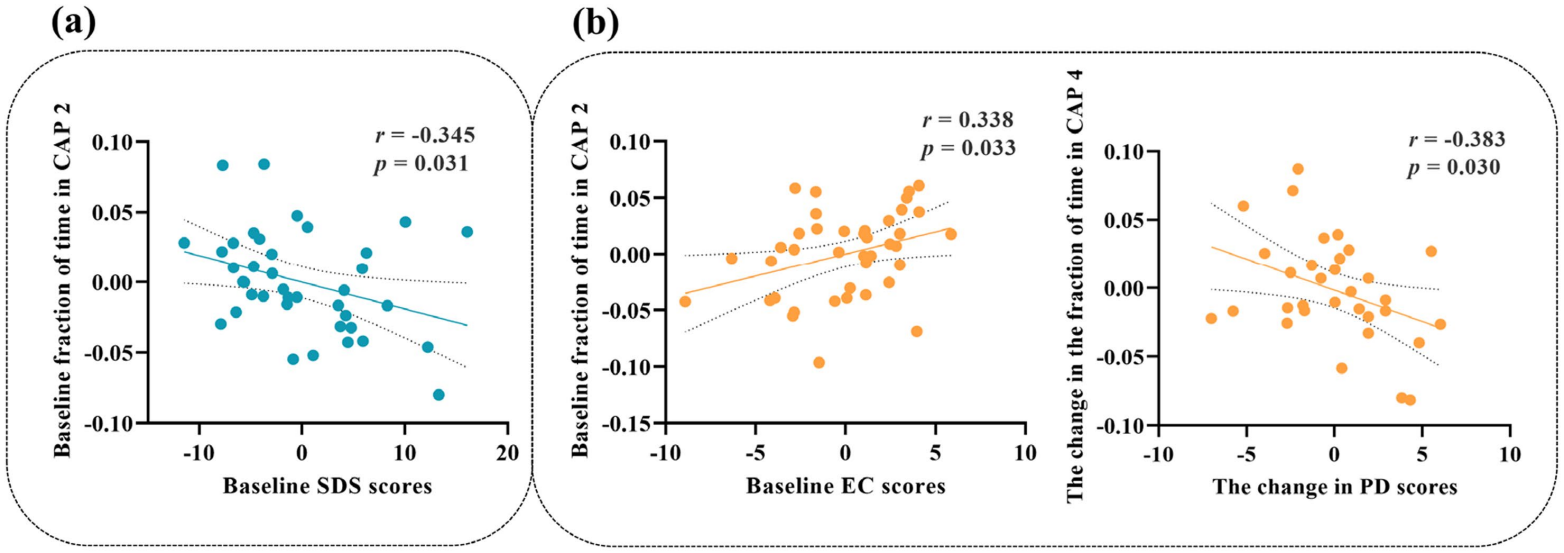
Correlations between behavioral variables and temporal metrics. Age, gender, and education level were controlled as covariates. The *x*- and *y*-axes represent residual behavioral variables and CAP temporal metrics after regressing out age, sex, and educational level. **(a)** Correlations observed in the sad group. **(b)** Correlations observed in the neutral group.

In the sad group, the baseline fraction of time in CAP 2 was negatively associated with the baseline SAS score (*r* = −0.345, *p* = 0.031) (Figure 5a). The change in the fraction of time in CAP 4 was negatively correlated with the change in PD scores (*r* = −0.383, *p* = 0.030) in the neutral group. In addition, the baseline fraction of time in CAP 2 was positively correlated with the baseline EC scores (*r* = 0.338, *p* = 0.033) (Figure 5b).

## 4 Discussion

By applying CAP analysis, we identified four reproducible co-activation patterns reflecting distinct large-scale network configurations. Notably, participants in the sad condition exhibited an increased post-stimulation engagement of CAP 2 (VN+, SMN+, FPN–) and a decreased engagement of CAP 4 (SMN+, SN+, DMN–) compared with the sham group, but not the neutral condition. In addition, the transition probability from CAP 2 to CAP 3 showed significantly greater increase in the sad condition than in the neutral condition. Taken together, these results suggest that the brain state during stimulation affects the effects of right VLPFC-targeted rTMS on brain dynamics, providing evidence consistent with state-dependent effects.

After rTMS, the sad group exhibited a significantly greater CAP 2 fraction of time, suggesting that the emotional context biases the direction of network reconfiguration during VLPFC inhibition. CAP 2 reflects a network configuration characterized by co-activation of the VN and SMN, along with deactivation of the FPN. This pattern may reflect a brain state characterized by externally oriented perceptual processing and reduced top-down cognitive control, which may correspond to greater attentional focus on external sensory input. Prior studies have shown that sensory processing and attention can be modulated by the affective significance of stimuli and are often enhanced by negative emotion (Vuilleumier, 2005). Moreover, the target VLPFC is involved in both reactive and regulatory tasks, maintaining bidirectional connections with the amygdala and sensory cortices (Pessoa, 2008; Ray and Zald, 2012). It also shows strong associations with areas linked to emotional expression, such as the frontal pole, as well as regions responsible for emotional regulation, including the dorsolateral prefrontal cortex (Kohn et al., 2014; McTeague et al., 2020). In our prior study conducted on the same sample, which relied on static measures of brain activity, we observed that rTMS elicited increased amplitude of low-frequency fluctuations in the right superior occipital gyrus and decreased amplitude of low-frequency fluctuations in the right middle frontal gyrus in the sad group compared with the neutral group (Hou et al., 2025). Our results suggest that exposure to sad emotional context during VLPFC inhibition shifts the brain toward a perceptually driven state, which may reflect a relative weakening of top–down regulatory processes.

In contrast, following rTMS of the VLPFC, participants who viewed the sad film exhibited a reduced fraction of time in CAP 4. CAP 4 is characterized by co-activation of the SN and SMN, coupled with deactivation of the DMN. This pattern may reflect a state characterized by reduced self-referential processing and increased salience detection, consistent with enhanced responsiveness to external salient information. The SN is critically involved in detecting emotionally relevant information and orchestrating rapid switching between internal and external attention states (Seeley et al., 2007; Sridharan et al., 2008). Previous studies have shown that the VLPFC, anterior insula, and presupplementary motor area together constitute key nodes of the multiple demand network, which supports domain-general cognitive control (Duncan, 2010; Fedorenko et al., 2013; McTeague et al., 2020). Beyond cognitive control, the VLPFC and its interactions with subcortical affective regions, primarily the insula and amygdala, play a crucial role in voluntary emotion regulation (Ochsner et al., 2012; Sridhar et al., 2024). Moreover, we found that changes in the fraction of time in CAP 4 were negatively associated with changes in PD scores in the neutral group. Previous evidence showed that rTMS stimulation of the VLPFC modulates activity in the insula and other prefrontal regions, thereby causally contributing to emotion regulation (He et al., 2023a). Therefore, these findings suggest that rTMS targeting VLPFC during sadness may influence neural systems involved in salience detection and emotion regulation.

The observation that the neutral group exhibited a minimal or absent change further reinforces the state dependence of rTMS on brain dynamics. Accumulating evidence increasingly converges on the view that rTMS interacts with the brain’s ongoing functional state at the time of stimulation (Bradley et al., 2022). Accordingly, the oscillatory brain state, the cognitive brain state, and the recent history of the brain state of individuals may critically affect the effects of rTMS (He et al., 2025; Sack et al., 2024). For instance, Scangos et al. (2021) implanted multi-site intracranial electrodes in a severely depressed patient and demonstrated that stimulation-evoked responses varied depending on the patient’s symptom state at the time of stimulation. Similarly, Isserles et al. (2011) reported that negative cognitive–emotional reactivation during deep TMS in patients with depression affects therapeutic outcome. In addition, chronometric TMS-fMRI studies have shown that the brain state can influence local and remote effects of TMS (Grosshagauer et al., 2024). Taken together, these findings converge with the present results, suggesting that the neuromodulatory impact of rTMS is significantly influenced by the brain state at the time of stimulation.

There are several limitations in this study. First, the study was conducted in a healthy sample, which may limit the generalizability of the findings to clinical populations. The rTMS state-dependent effects on brain dynamics observed in healthy individuals may differ from those in patients with major depressive disorder, who often exhibit chronic alterations in neuroplasticity and large-scale brain network organization. Future studies are needed to replicate and extend these findings in clinical populations. Second, the absence of a sham stimulation condition under the neutral emotional context limits the ability to fully disentangle the main effects of stimulation and emotional context, as well as their interaction. Although our results provide evidence consistent with state-dependent effects of rTMS, this incomplete factorial design precludes definitive conclusions. Future work employing a fully crossed factorial design (active vs. sham; sad vs. neutral) is needed to more precisely clarify these effects.

## 5 Conclusion

In conclusion, we found that participants exposed to sad emotional states exhibited pronounced alterations in CAP dynamic metrics following stimulation compared with the sham group, whereas only minimal effects were observed in the neutral condition. Collectively, these findings suggest that the neuromodulatory effects of right VLPFC-targeted rTMS may be influenced by the emotional context during stimulation. This study provides evidence consistent with state-dependent effects of rTMS and highlights the important role of emotional context in modulating these effects.

## Data Availability

The raw data supporting the conclusions of this article will be made available by the authors, without undue reservation.

## Footnotes

1
http://www.fil.ion.ucl.ac.uk/spm/

2
http://rfmri.org/DPARSF
