# Supplementary material for: State-dependent modulation of brain co-activation patterns induced by individually targeted VLPFC stimulation during viewing emotional film clips
Source: Front Hum Neurosci. 2026 May 12;20:1824753. doi: 10.3389/fnhum.2026.1824753 (PMC13201408; doi:10.3389/fnhum.2026.1824753)
Supplement: Supplementary file 1 [file Data_Sheet_1.docx]

**State-dependent Modulation of Brain Co-activation Patterns Induced by Individually Targeted VLPFC Stimulation during Viewing** **Emotional Film Clips**

supplementary materials

**Supplemental Results**

To verify the effectiveness of the emotional induction in the present study, participants provided subjective ratings of their emotional experience following film viewing, including sadness, pleasure, arousal, and dominance. The results confirmed that the sad condition elicited significantly higher mean sadness and arousal ratings across the five days compared to the neutral condition. Specifically, a one-way ANOVA revealed a significant group effect on both sadness and arousal ratings (*F* = 118.45, *p* < 0.001; *F* = 19.20, *p* < 0.001). Post hoc comparisons revealed that the sad group and the sham group exhibited significantly higher sadness and arousal ratings compared with the neutral group (*p* < 0.001), whereas no significant differences were observed between the sad group and the sham group (Figure S2). These findings support the validity of the emotional induction.

To further assess the robustness of the clustering solution, we re-derived the CAP states using only the pre-session data and compared them with those obtained from the full dataset. The spatial patterns of the CAPs showed a high degree of similarity, with correlation coefficients of 0.97, 0.98, 0.98, and 0.97 for the four CAPs, respectively. These results indicate that the identified CAP structure is highly stable (Figure S3).

**Supplemental Figures**

**
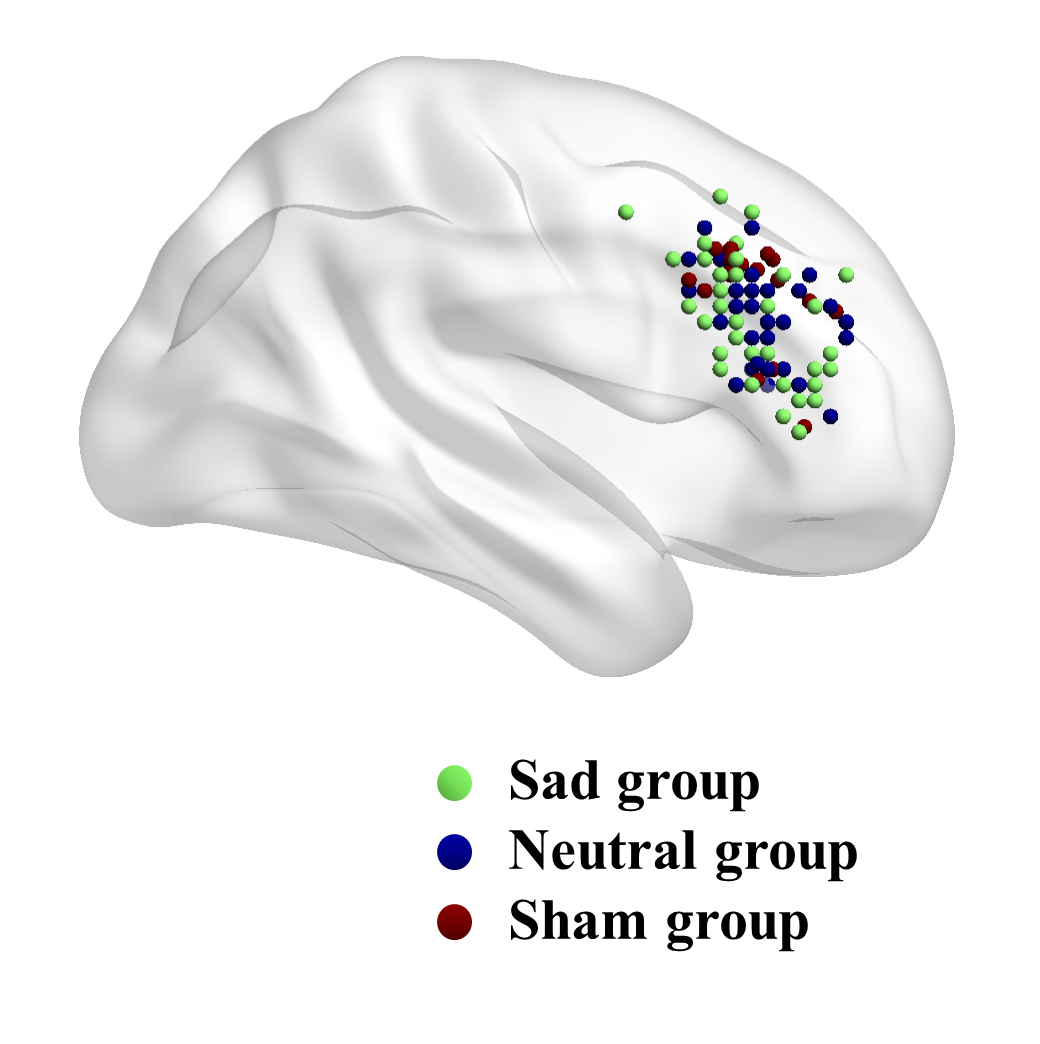
**

FIGURE S1 Individual TMS targets coordinates. A visualization of the individualized stimulation coordinates for each participant in three groups in standard space.


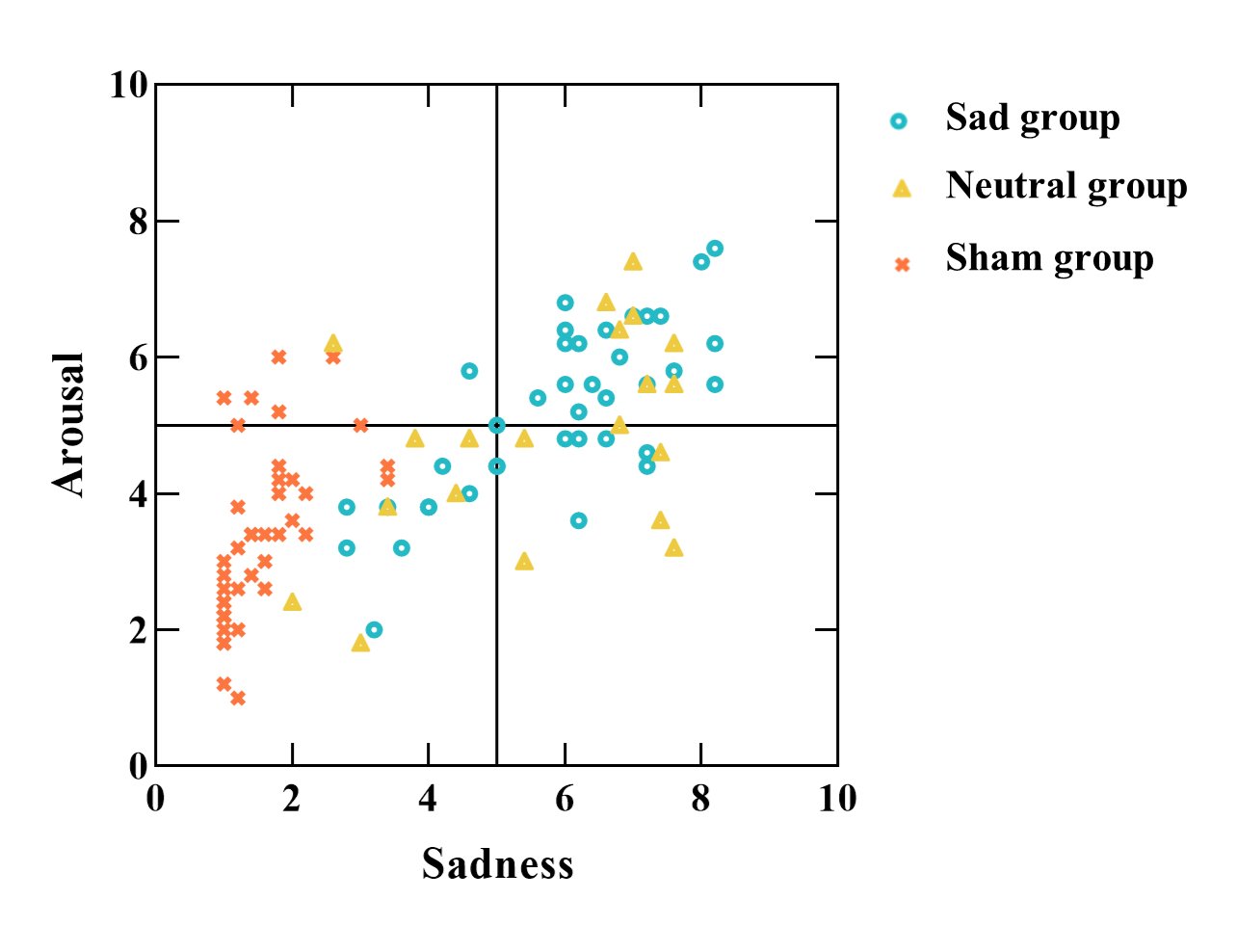


FIGURE S2 The distribution of the film clips in the affective space (mean values for sadness on the horizontal axis and arousal on the vertical axis).


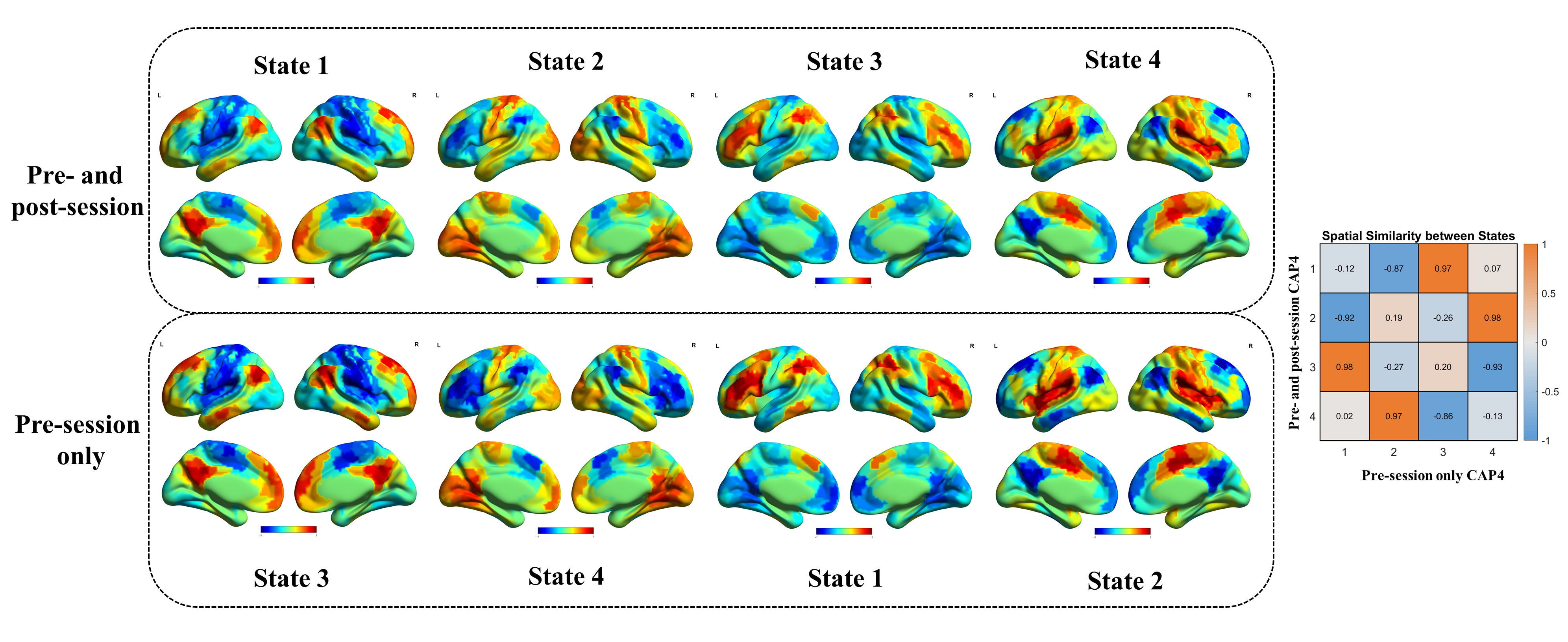


FIGURE S3 Spatial similarity of CAP states derived from the pre-session data and the full dataset.
